# Notes on the genus *Indocnemis* Laidlaw, 1917 in Vietnam with description of *Indocnemismarijanmatoki* sp. n. (Odonata, Zygoptera, Platycnemididae)

**DOI:** 10.3897/zookeys.809.29058

**Published:** 2018-12-19

**Authors:** Quoc Toan Phan

**Affiliations:** 1 Center for Entomology and Parasitology Research, Institute of Research and Training of Medicine, Biology and Pharmacy, Duy Tan University, 3 Quang Trung, Da Nang, Vietnam Duy Tan University Da Nang Vietnam

**Keywords:** *Indocnemis*, new species, Odonata, Platycnemididae, Vietnam

## Abstract

*Indocnemismarijanmatoki***sp. n.** (holotype ♂, 12°07'10.0"N, 108°5'51.0"E, 1503 m a.s.l., Hon Ba Nature Reserve, Nha Trang city, Khanh Hoa Province, central Vietnam) is described based on both sexes. The morphological variation of *Indocnemisorang* (Förster in Laidlaw, 1907) is discussed and its distribution in Vietnam updated.

## Introduction

Laidlaw (1917) established the genus *Indocnemis* characterized it as having the “wing relatively broad and rounded, [with] 3 cells between the quadri-lateral and sub-nodal. Reticulation on the fore-wing not so dense (not more than 250 cells on the hind-wing)”. This was based on a single male of *Indocnemiskempi* from Assam, India, and according to Laidlaw (1917), this male has a blue antehumeral stripe on the synthorax and the appendages are entirely black. In 1917, Laidlaw thought that *I.kempi* might be congeneric with *Trichocnemisorang* Förster, 1907. In 1931, he transferred *T.orang* to the genus *Indocnemis* after examining the type specimen and several other specimens of from Malaysia and Thailand (Laidlaw 1931); he also suggested that his Assamese *I.kempi* might be a synonym of *I.orang*. He wrote that “young males appear to have a narrow blue band on either side of the dorsum of the synthorax”, but “in adult male, the blue stripe of the dorsum of the synthorax seems to widen considerably, so as to form a large oblong-oval mark extending inwards almost to the mid-dorsal carina”. The change from a narrow dorsal tripe on the synthorax in young to a large shield in adult specimens has never been confirmed in the field in Vietnam, where immature males have similar large shields as adults, but of a pale yellow colour (Kompier in. litt.). Moreover, no case of expanding pale elements of the pattern with age are known in Odonata, although their reduction with age is common. Therefore, it seems possible that Laidlaw (1931) based his discussion on specimens of both forms. It requires further examination of the Malaysian and Thai specimens used in Laidlaw’s (1931) description to establish whether there truly is a change in these characters with maturation. Asahina (1985) downgraded *I.kempi* to a synonym of *I.orang* and showed that males from Assam have a narrow stripe on the dorsum of the synthorax (Asahina 1985: 9, fig. 30). However, in 1997 Asahina stated “Now I am changing my previous idea (1985a), in which *kempi* (1917) was synonymized with *orang* (1907), though the former was recognized as a large-sized form of the latter.” Asahina (1997) concluded that the genus *Indocnemis* consisted of only one species, *I.orang*, which has two forms differing in size: the first form (forma orang), originally described from Perak, Malaysia (measurements of males: abdomen 46–47 mm, hind wing 32–34 mm) is smaller than the second form (forma kempi, based on specimens from Assam and Tam Dao National Park, northern Vietnam) (measurements of males: abdomen 51–57 mm, hind wing 35–38 mm) (Laidlaw 1917, 1931; Asahina 1985, 1997). Indocnemsorangformakempi in Tam Dao also has a broader thoracic stripe than the population in Cuc Phuong (Asahina 1997).

Dijkstra et al. (2013) pointed out that the two genera *Indocnemis* and *Coeliccia* Kirby are paraphyletic. *Indocnemis* differs from *Coeliccia* by the anal crossing (Ac) ending on the anal bridge vein, not on the wing margin (Asahina 1997). However, several specimens in my collection of, for instance, *Coelicciacyanomelas* (Fig. 35) do not have the Ac ending at the wing margin, just like *Indocnemis* (see also Wilson and Reels 2003). Therefore, this character cannot be used to consistently separate the two genera. Wilson and Reels (2003) provided another character of the genus *Indocnemis*: the presence of four cells between the discoidal cell and the nervure descending from the subnode, whereas *Coeliccia* has just three cells. However, some *I.orang* specimens have only three cells (Fig. 32) and some *Coeliccia* species have four cells (Fig. 33) or two cells (Figs 34–35). Wilson and Reels (2003) stated that this character was also variable and that some *ambigua* only had three cells. Therefore, these venational characters are variable, even within a species, and there is no basis on which to distinguish the genera *Indocnemis* and *Coeliccia.* Although Wilson and Reels (2003) transferred *Coelicciaambigua* Asahina, 1997 to the genus *Indocnemis*, their interpretation has not been accepted in several later publications (e.g. Phan and Kompier 2016; Kosterin and Kompier 2017; Phan and Tran 2018). Moreover, the genital ligula of *C.ambigua* is structurally simple and unlike the two *Indocnemis* species discussed here. Therefore, I retain the original combination of *C.ambigua* and do not list this species in the genus *Indocnemis* in this paper.

I do not intend here to synonymize the genus *Indocnemis* with *Coeliccia*, but I do not think the current distinction is valid (see also Dijkstra et al. 2013). The taxonomic relationships between these two genera may be solved in the future based on further molecular analysis. Here I characterise the morphological variation of the widespread species *I.orang* in Vietnam and describe a second member of the genus, *I.marijanmatoki* sp. n. I place the new species in the genus *Indocnemis* in view of its great similarity to *I.orang* by the structure of the appendages and the genital ligula of the male, and the body coloration of both sexes. *Indocnemismarijanmatoki* sp. n. differs from *I.orang* by the shape of its cerci and female prothorax structures.

## Material and methods

Specimens of *Indocnemisorang* used for comparing with the new species were collected on the same date and location, Hon Ba Nature Reserve of Khanh Hoa Province (km 19, 12°06'49.3"N, 108°59'37.3"E, 418 m a.s.l.) as the types of *I.marijanmatoki* sp. n. The habitus of holotype and the female paratype were photographed with a Nikon D3300 digital camera and Nikon AFS DX Micro Nikkor 85 mm f/3.5G ED VR lens. Photographs in nature were taken with a Nikon D3300 digital camera with Nikon AF Micro 200 mm f4D IF-ED lens. Other colour photographs were taken with an Axiocam Erc 5s camera on Zeiss Stemi 508 stereomicroscope. Illustrations were made with Adobe Photoshop 7.0.

Morphological nomenclature used for damselfly structures follows Phan and Kompier (2016). Preparation of specimens follows standard practice as for instance described in Paulson (2018).

Abbreviations:

**S1–10** abdominal segments 1 to 10;

**Px** postnodal crossveins;

**HW** hindwing;

**FW** forewing;

**a.s.l** above sea level.

## Results

All examined mature males of *Indocnemisorang* have synthoracic dorsal stripes covering most of mesepisternum and black cerci with blue marks dorsally (Fig. 24), that agree with the forma kempi, but only specimens from Bach Ma National Park have the same large body size (male: abdomen 55–57 mm, hind wing 37–38 mm) of this form as described by Asahina (1997). Measurements of others specimens fall within the size range of I.orangformaorang (male: abdomen 46–50 mm, hind wing 35–36 mm). As the males of Vietnamese populations of *I.orang* display considerable variation in body size among different individuals, this alone does not seem a sound basis to divide them into two forms as in Asahina’s interpretation. Most of examined males of Vietnamese *I.orang* in this paper are similar to those in photos of *I.orang* taken in Malaysia by Choong (2018), Meghalaya, India by Joshi et al. (2018) or Thailand by Farrell (2018) in respect of the large dorsal shield on the synthorax and very dark cerci, which are entirely black or black with blue mark dorsally. Wilson and Reels (2003) also pointed out that Asahina’s (1997) treatment did not make it clear whether Vietnamese specimens of *I.orang* should be assigned to formae *orang* or *kempi*. There is the population, probably unique, of *I.orang* in Cuc Phuong that is consistent with the original description of I.orangformakempi in having its synthorax displaying a dorsal stripe, not a shield (Fig. 9) although its cerci are pale yellow (Fig. 10). Therefore, I maintain the division of Vietnamese *I.orang* into two forms on the basis of the difference of the body coloration pattern of the mature male as follows:

Indocnemisorangformaorang: Large shield-shaped stripe on synthorax (Figs 8, 23), cerci black with blue marks dorsally (becoming entirely black after acetone treatment) (Fig. 24). Throughout the species’ range.

Indocnemisorangformakempi: Narrow stripe on synthorax (Fig. 9), cerci pale yellow (Fig. 10). Cuc Phuong National Park.

The population of *I.orang* in Tam Dao, as reported by Asahina (1997), also should be transferred into the forma orang (not *kempi*) based on the shield-like oval mark on the synthorax (Asahina 1997: 33, figs 66, 67).

### Indocnemis orang (Förster in Laidlaw, 1907) forma orang

Taxon classificationAnimaliaOdonataPlatycnemididae

Figures 1–4 , 8 , 17–20 , 23 , 24 , 29 , 30 , 32

#### Examined specimens.

1 mature male, Pia Oac National Park, Cao Bang Prov., 16 May 2015; 1 mature female, Xuan Son National Park, Phu Tho Prov., 15 September 2015; 1 mature female, Vu Quang National Park, Ha Tinh Prov., 07 April 2015; 6 mature males, 5 mature females, Bach Ma National Park, Thua Thien Hue Prov., 27 June 2017; 1 mature male, 1 mature female, Sao La Nature Reserve, A Luoi District, Thua Thien Hue Prov., 18 September 2015; 1 mature male, 3 mature females, Deo Lo Xo, Phuoc Son District, Quang Nam Province, 05 August 2017; 3 mature males, 1 mature female, Nam Giang District, Quang Nam Prov., 25 May 2017; 1 mature male, 1 mature female, Bhalee, Tay Giang District, Quang Nam Prov., 18 September 2015; 1 mature male, 2 mature females, Ba Na Nature Reserve, Da Nang city, 25 May 2015; 1 mature male, Kon Chu Rang Nature Reserve, Gia Lai Prov., 11 March 2017; 1 mature male, Dak Roong, K’Bang District, Gia Lai Prov., 24 May 2018; 1 mature female, Chu Mom Ray National Park, Kon Tum Prov., 22 May 2017; 3 mature males, 1 mature female, Chu Yang Sin National Park, Dak Lak Prov., 19 May 2018; 1 mature male, Mang Canh, Kon Plong District, Kon Tum Prov., 22 September 2015; 1 mature male, 1 immature male, Maria pass, Bao Loc District, Lam Dong Prov., 16 March 2016; same location, 1 immature male, 3 mature female, 22 April 2016; 1 mature male, same location, 11 May 2017; 3 mature males, 1 immature male, 1 immature female, km19, Hon Ba Nature Reserve, Khanh Hoa Prov., 16 April 2017; 1 mature female, same location, 08 May 2015. All materials were collected by the author. 2 mature females, Kon Ka Kinh National Park, Gia Lai Prov., 6 April 2018, To Van Quang leg.

#### Remarks.

**Figures 1–6.**
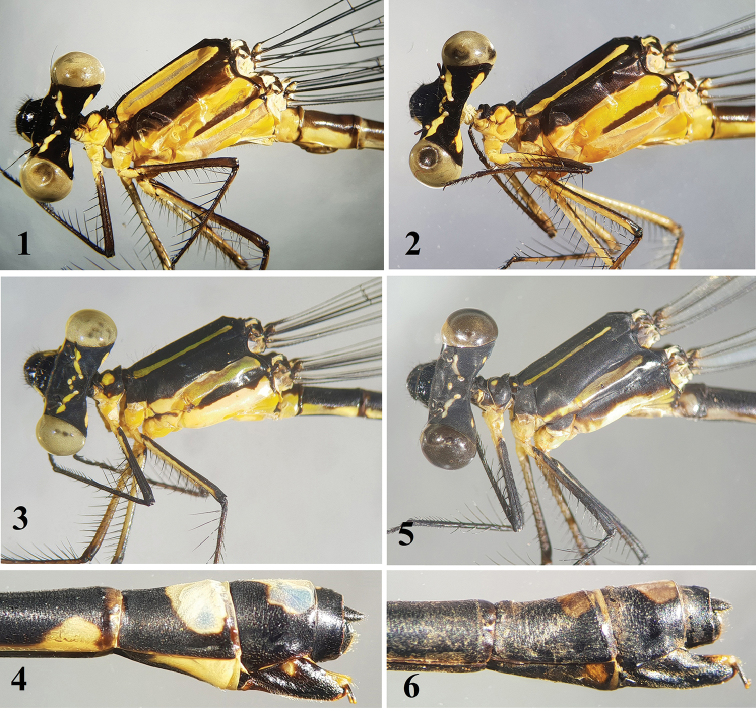
*Indocnemisorang*, **1–4** forma orang & **5, 6** forma kempi. **1** immature male, Hon Ba **2** immature female, Hon Ba **3, 4** head, thorax and abdominal tip of mature female, Phuoc Son **5, 6** head, thorax and abdominal tip of mature female, Cuc Phuong.

All examined immature males of Indocnemisorangformaorang differ from the mature specimens by the following characters: middle lobe of prothorax is mostly yellowish (Fig. 1) but this mark reduced to a very small dot on either side or absent in mature males (Fig. 23); large dorsal shield on synthorax yellow, not purple as in mature males (Fig. 1); metepimeron entirely yellowish (Fig. 1) but largely black in mature males (Fig. 23); dorsal S9–10 and whole appendages pale yellowish, whereas cerci are black with blue marks dorsally in mature males (Fig. 24). The immature females are very similar to mature ones except that dorsal head stripes and yellow spots in either side of middle lobe of prothorax broader and antehumeral stripe is yellow, not blue as in mature females (Figs 2, 3).

### Indocnemis orang (Förster in Laidlaw, 1907) forma kempi

Taxon classificationAnimaliaOdonataPlatycnemididae

Figures 5 , 6 , 9 , 10

#### Examined specimens.

1 mature male, 1 mature female, Cuc Phuong National Park, Ninh Binh Prov., 25 June 2018, Q.T. Phan leg.

#### Remarks.

Asahina (1997) did not describe the colour of the dorsal stripes on synthorax of his *orang* forma kempi from Cuc Phuong. I can now confirm that the dorsal stripe of the mature living male of this form in Cuc Phuong is blue (Fig. 9). Asahina (1997) also did not mention the pale yellowish appendages of the males from Cuc Phuong (Fig. 10). Females of the two forms *orang* and *kempi* can be separated by their body pattern: the yellow spot on the middle lobe of prothorax and lateral stripes on synthorax in forma kempi are smaller (Fig. 5) than those in forma orang (Fig. 3); the bluish markings on dorsal S9–10 in forma kempi are smaller and in forma orang; and S8 is black, without yellow marks as in forma orang (Figs 4, 6). In female forma kempi in Cuc Phuong, the structure of the posterior lobe of the prothorax is the same as in the female of forma orang.

#### Distribution.

*Vietnam*: Vinh Phuc (Tam Dao National Park), Ninh Binh (Cuc Phuong National Park), Thua Thien Hue (Bach Ma National Park), Lam Dong (Bao Loc District) [Do and Dang 2007], Ha Noi (Ba Vi National Park) [Kompier 2018], Cao Bang (Pia Oac National Park), Phu Tho (Xuan Son National Park), Ha Tinh (Vu Quang National Park), Quang Nam (Phuoc Son and Tay Giang Districts), Gia Lai (Kon Chu Rang Nature Reserve and Kon Ka Kinh National Park), Dak Lak (Chu Yang Sin National Park), Kon Tum (Kon Plong District and Chu Mom Ray National Park) and Khanh Hoa Provinces (Hon Ba Nature Reserve); *Laos*: Luang Prabang, Oudomxay and Xiang Khouang Provinces [Yokoi and Souphanthong 2014]; *Thailand*: Khao Ram Rome Moutain, Nakhon Si Thammarat Province [Laidlaw 1931], Petchaburi (Nam Nao National Park), Phang Nga (Khao Lak) and Chantaburi Provinces (Khao Soi Dao National Park) [Noppadon Makbun pers. comm.]; *China*: Fujian, Guangdong, Fukien, Sichuan and Guangxi Provinces [Asahina 1985, Wilson and Reels 2003]; *Malaysia*: Perak [Laidlaw 1931] and Cameron Highlands [Asahina 1985]; *India*: Assam and Sikkim [Asahina 1985, Laidlaw 1917], *Bangladesh*: ? [Subramanian 2010].

### Indocnemis marijanmatoki sp. n.

Taxon classificationAnimaliaOdonataPlatycnemididae

http://zoobank.org/83FEE544-10B1-4754-A678-9ACD521AF85D

Figures 7 , 11 , 12 , 13–16 , 21 , 22 , 25–28 , 31

#### Type specimens.

**Holotype.** A mature male, folded wings in triangular envelope. Original label: “*Indocnemismarijanmatoki* sp. n., Hon Ba Nature Reserve, Nha Trang city, Khanh Hoa Province, Vietnam (12°07'10.0"N, 108°5'51.0"E, 1503 m a.s.l.), T.odo.16041705, Q.T. Phan leg”, “HOLOTYPE” [red handwritten label]. **Paratypes.** 1 mature male, 1 mature female, same date, location and collector as the holotype. All type specimens are deposited in the Zoological Collection of Duy Tan University, Da Nang city, Vietnam.

**Figures 7–10.**
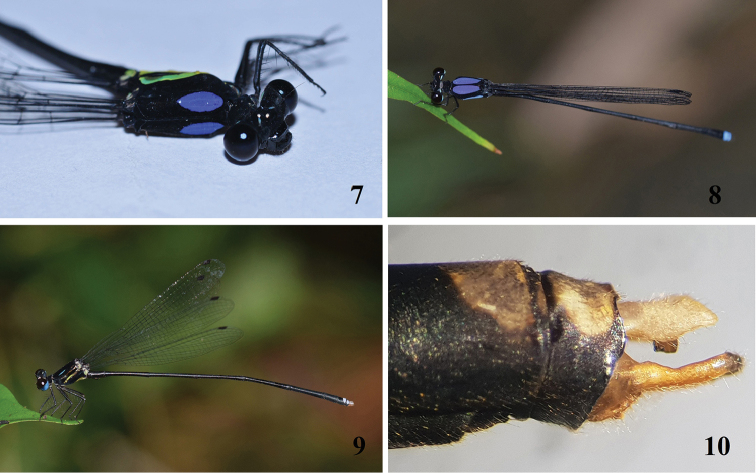
*Indocnemis* spp. **7***I.marijanmatoki* sp. n., ♂ from Kon Ka Kinh National Park, photographed by Mr To Van Quang **8***I.orang* ♂ in nature, Nam Giang, Quang Nam **9, 10** Habitus and appendages of *I.orang* ♂, Cuc Phuong National Park, photographed by the author.

#### Other specimens examined.

Two mature males, collected in a small stream on the main route to the top of the Kon Ka Kinh Mountain (14°19'83.5"N, 108°24'31.9"E, 1450 m a.s.l), Dak Hro village, Dak Roong commune, K’Bang District, Gia Lai Province, 09 April 2018, To Van Quang leg.

#### Etymology.

*Marijanmatoki*, a noun in the genitive case, after Marijan Matok (born 28 March 1972) of Ulm-Söflingen, Germany, in appreciation of his support of the author’s odonatological research in Vietnam through the International Dragonfly Fund.

#### Diagnosis.

The new species differs from *I.orang* with a combination of the following characters: in the male, the marking on dorsum of synthorax is small, shield-shaped; S9–10 entirely black; cerci short, of the length as S10, without a robust basal spine; paraproct entirely black. In the female, the posterior pronotal lobe of the prothorax is rather small, semicircular-shaped.

#### Description of holotype.

*Head* (Fig. 21). Labrum, genae, mandible and postclypeus shining black; anteclypeus dark brown. Antennae black except paler apical part of first and second segments. Top of head matte black with two long stripes adjacent to median ocellus running towards the base of the antennae and two water drop-shaped yellow spots just posterior to postoccipital lobes.

*Thorax* (Fig. 21). Prothorax entirely black. Synthorax black with a large oval-shaped marking and another tiny stripe in mesepisternum. The large marking blue in life, but becoming pale yellow surrounding a smaller blue part after acetone treatment. Mesepimeron black, metepisternum black with a large yellow stripe adjacent to mesocoxa and covering spiracle, interrupted before end of segment. A large yellow marking covering most of metepimeron.

*Legs* (Fig. 21). Coxae pale brown. Femora and tibiae black. Tarsi and armature brown.

*Wings* (Fig. 31) hyaline with black venation, 24 and 20 Px in FW and HW, respectively. Pterostigma brown, covering 2 underlying cells.

**Figures 11–12.**
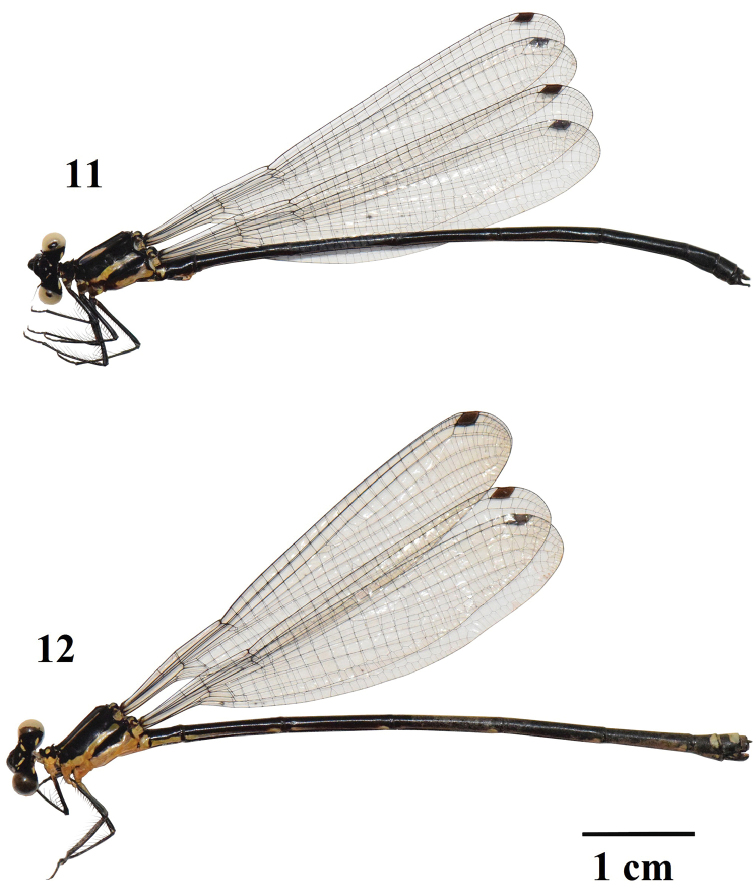
Habitus of *Indocnemismarijanmatoki* sp. n. **11** Holotype male **12** Paratype female.

*Abdomen* (Figs 11, 22). Segments entirely black excluded a large yellow marking laterally in S1 and ventral yellow line on S2 and a small whitish lateral spot on S10.

*Genital ligula* (Fig. 16) structurally simple with two long flagella.

**Figures 13–20.**
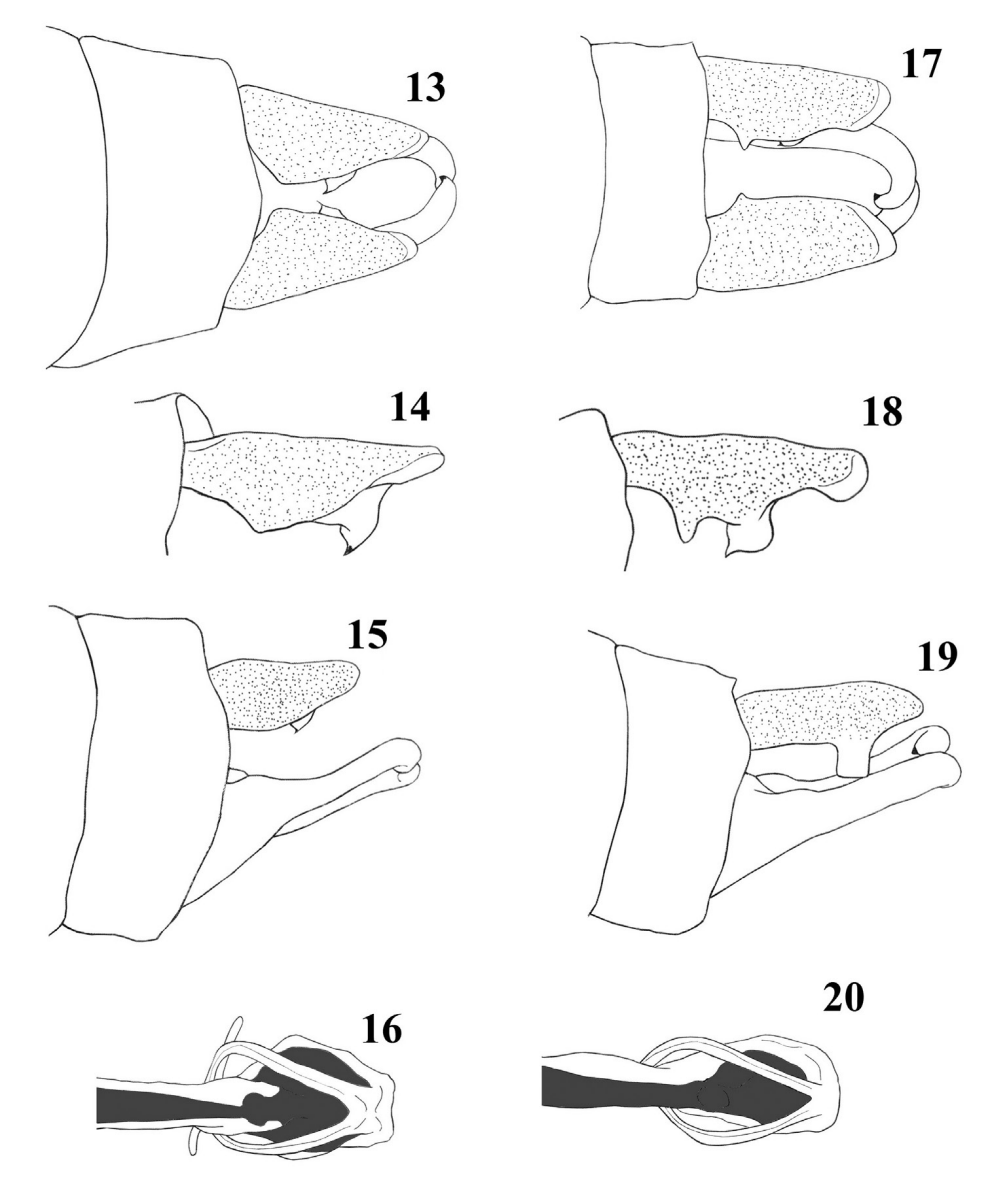
*Indocnemis* spp. ♂. **13–16***I.marijanmatoki* sp. n., holotype ♂ and **17–20***I.orang* (km19, Hon Ba Nature Reserve) **13, 17** appendages, dorsal view **14, 18** right cerci, oblique-dorsal view **15, 19** appendages, lateral view **16, 20** genital ligula, dorsal view.

*Anal appendages* (Figs 13–15, 22) black, except for dorso-apical margin of cerci, which are pale yellow. Cerci bearing a large ventral tooth near the apical portion. In lateral view, cerci as long as S10; in dorsal view, cercus narrowing distally and slightly pointed at apex. Paraproct longer than cercus, its tip directed medially and ending in a black tooth.

*Measurements*. HW 41 mm; abdomen (incl. appendages) 55 mm.

#### Variation in paratype male.

The paratype male differs from the holotype as follows: the blue marking on the mesepisternum slightly larger; the yellow marking in metepimeron not extending to the margin of metinfraepisternum as in the holotype; ventro-lateral S2 without yellow band and the pale marking on S10 bigger than in the holotype. In one male from Kon Ka Kinh National Park, cerci longer than S10, reaching the level of paraproct as in *I.orang*. Measurements ranges of hind wing 40 mm and abdomen (incl. appendages) 52 mm.

#### Description of female.

*Head* (Figs 25, 26). Labrum and postclypeus shining black; anteclypeus brownish; mandible and genae yellow, the lower margin of genae black. Dorsal head side matt black, ocelli pale yellow, there are two long stripes adjacent to ocellus and nearby two oval yellow spots. Posterior side of head black with two yellow spots as in male.

*Thorax* (Figs 12, 27, 28). Prothorax black, except two large oval spots at sides of middle pronotal lobe of pronotum; lower part of propleuron yellowish. Posterior pronotal lobe well developed, but only half as wide as middle lobe, rounded (Figs 27, 28). Mesepisternum black with a long and narrow antehumeral stripe; mesepimeron black, metepisternum black with a large yellow stripe, rounded at the end and covering spiracle and metathoracic cross sutures; this mark connected to yellow part of metepimeron and metinfraepisternum.

*Legs.* Coxae and trochanter yellowish. Femora black with yellow marks at base. Tibia, tarsus and armature black.

*Wings.* Hyaline, 23–24 and 20 Px in FW and HW, respectively. Pterostigma brown, covering 1.5–2 cells.

*Abdomen* (Fig. 12). S1 black with a large lateral yellow spot; S2–3 with a yellow latero-ventral band; S4 with two tiny yellow spots at segment margins; S5–7 with a tiny yellow spot at ventral-apical margin of each segment; S8–9 black with a large bluish marking dorso-apically on each segment; S10 black. Cerci black, ovipositor black with small yellow spot anteriorly and dorsally at apex.

*Measurements*. HW 41 mm; abdomen (incl. appendages) 55 mm.

#### Habitat and ecology.

At the type locality, the new species was found at a narrow (2–3 m wide), shallow stream with sandy bottom. Specimens were collected in April, which otherwise is early for other dragonflies and damselflies, so only *Anotogaster* sp. was found at the same stream. At the two localities where the new species was found, *I.marijanmatoki* sp. n. and *I.orang* occur at quite different elevations. The new species occurs at very high elevations, from 1,400–1,500 m a.s.l., while *I.orang* is usually found in the areas ranging from 300–600 m a.s.l.

#### Discussion.

**Figures 21–24.**
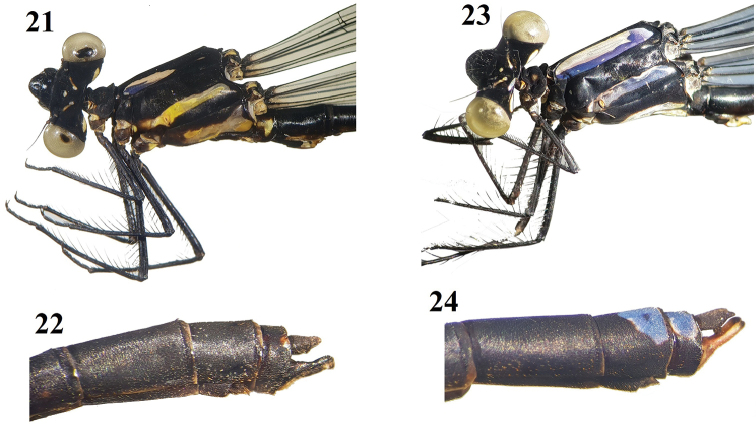
*Indocnemis* spp. ♂. **21, 22***I.marijanmatoki* sp. n., holotype ♂ **23, 24***I.orang* (km19, Hon Ba Nature Reserve). **21, 23** Head & thorax **22, 24** tip of abdomen.

In the male, the cerci of *Indocnemismarijanmatoki* sp. n. are relatively short, as long as S10 and lack a robust basal spine (Figs 13–15), while in *I.orang*, the cerci are 1.5 times the length of S10 and have a robust basal spine (Figs 17–19); the paraprocts of *I.marijanmatoki* sp. n. are entirely black (Fig. 22), but those of *I.orang* are yellowish (Fig. 24); the dorsum of S9–10 of *I.marijanmatoki* is black (Fig. 22), while strikingly marked with blue in *I.orang* (Fig. 24); and finally, the bluish dorsal stripe extends above the mesepimeron, covering most of the mesepisternum in *I.orang* (Figs 8, 23) but is reduced to a smaller shield-shaped mark and another tiny oval spot in *I.marijanmatoki* (Figs 7, 21). Females of both species are very similar in appearance but differ clearly in the shape of the posterior lobe of the prothorax. In *I.marijanmatoki* sp. n., this structure is prominent, but clearly less wide and semicircular in shape (Fig. 28), whereas it is much wider in *I.orang* (Fig. 30). The yellow stripe on the dorsum of the head of all examined specimens of *I.orang* extends to the margin of the compound eyes (Fig. 29), just like in Thai (Asahina 1985: 8, fig. 27) and Indian specimens (Asahina 1997: 9, fig. 32), while these are divided into two stripes, never touching the margin of the compound eye (Fig. 26) in *I.marijanmatoki* sp. n.

**Figures 25–30.**
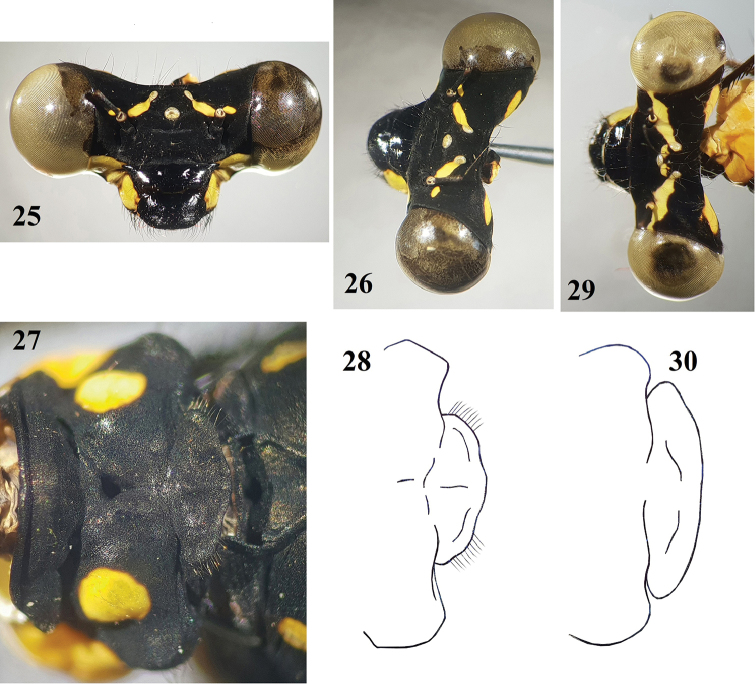
*Indocnemis* spp. ♀. **25–28***I.marijanmatoki* sp. n. and **29, 30***I.orang* (km19, Hon Ba Nature Reserve) **25** head, frontal view **26, 28** head, oblique-dorsal view **27** prothorax, dorsal view **29, 30** posterior lobe of prothorax, dorsal view.

**Figures 31–35.**
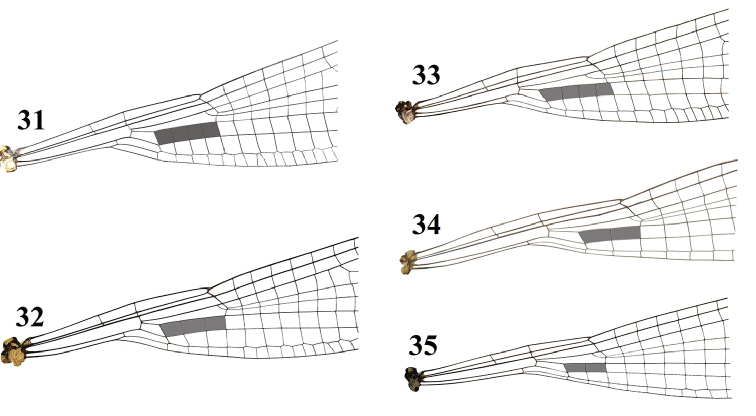
*Indocnemis* spp. and *Coeliccia* spp., base of hind wing. The brownish cells indicated the number of cells between discoidal cell and the nervure descending from the subnode **31***I.marijanmatoki* sp. n., holotype male **32***I.orang*, male (Km 19, Hon Ba Nature Reserve) **33***C.ambigua*, female (Ba Be National Park, Bac Kan Prov., 6.vii.2015, Hoang Vu Tru leg.) **34***C.mingxiensis*, male (Bach Ma National Park, Thua Thien Hue Prov., 27.vi.2017, Q.T. Phan leg.) **35***C.cyanomelas*, male (Bach Ma National Park, Thua Thien Hue Prov., 27.vi.2017, Q.T. Phan leg.).

**Figure 36.**
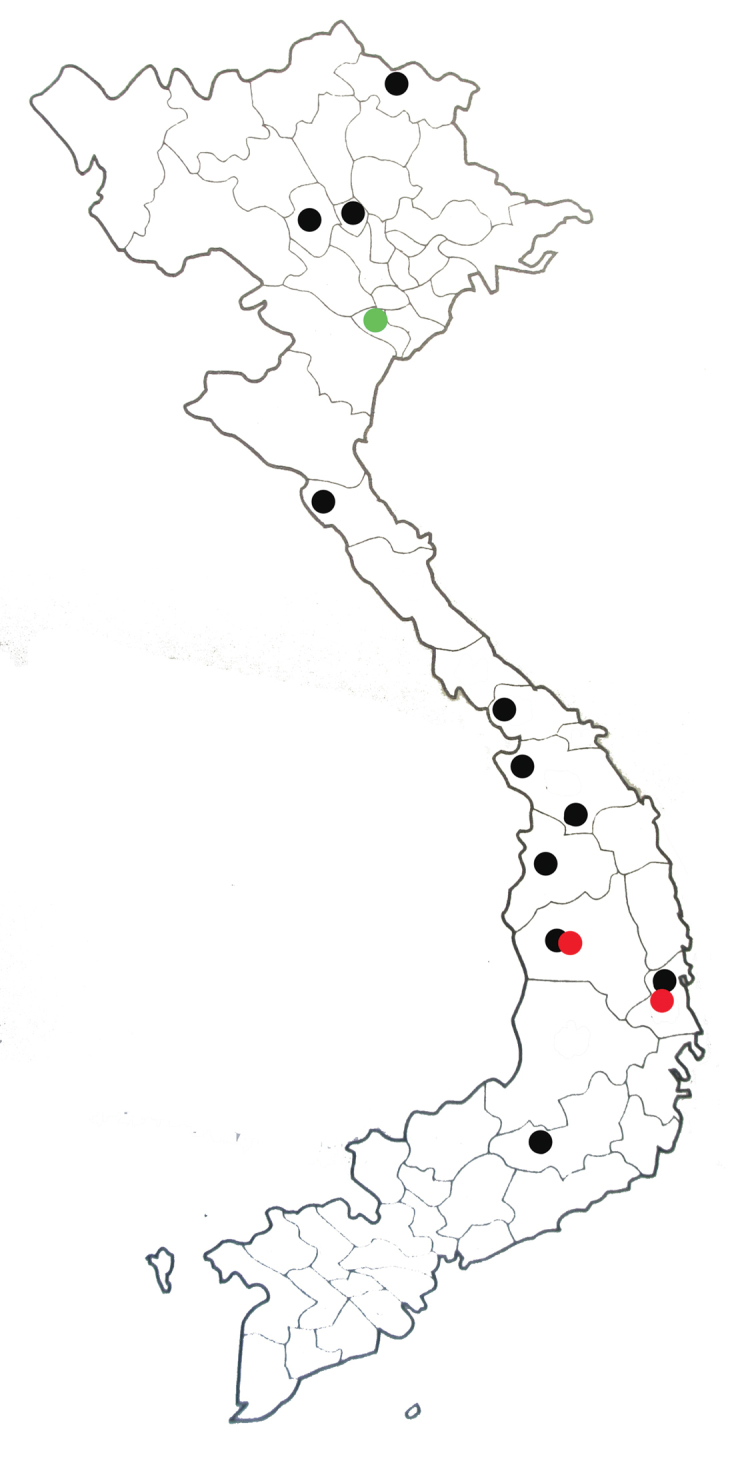
Provincial distribution map of *Indocnemismarijanmatoki* (**●**), I.orangformaorang (**●**) and I.orangformakempi (**●**) in Vietnam based on Do and Dang 2007) and this study.

## Supplementary Material

XML Treatment for Indocnemis orang (Förster in Laidlaw, 1907) forma orang

XML Treatment for Indocnemis orang (Förster in Laidlaw, 1907) forma kempi

XML Treatment for Indocnemis marijanmatoki
